# Epidemiology of hepatitis E virus infection in animals in Africa: a systematic review and meta-analysis

**DOI:** 10.1186/s12917-021-02749-5

**Published:** 2021-01-25

**Authors:** Abdou Fatawou Modiyinji, Jean Joel Bigna, Sebastien Kenmoe, Fredy Brice N. Simo, Marie A. Amougou, Marie S. Ndangang, Moise Nola, Richard Njouom

**Affiliations:** 1 Department of Virology, Centre Pasteur of Cameroon, Yaoundé, Cameroon; 2 Department of Biology and Animal Physiology, Faculty of Sciences, University of Yaoundé I, Yaoundé, Cameroon; 3 Department of Epidemiology and Public Health, Centre Pasteur of Cameroon, Yaoundé, Cameroon; 4 School of Public Health, Faculty of Medicine, University of Paris Sud, Le Kremlin-Bicêtre, France; 5 Department of Medical Information and Informatics, Rouen University Hospital, Rouen, France

**Keywords:** Hepatitis E, Epidemiology, Africa, Animals, Veterinary

## Abstract

**Background:**

Hepatitis E virus (HEV) is a major cause of acute hepatitis in humans worldwide and have high burden in the resource-limited countries. Better knowledge of the epidemiology of hepatitis in animals in Africa can help to understand the epidemiology among humans. The objective of this study was to summarize the prevalence of HEV infection and distribution of HEV genotypes among animals in Africa.

**Methods:**

In this systematic review and meta-analysis, we comprehensively searched PubMed, EMBASE, African Journals Online, and Africa Index Medicus from January 1st, 2000 to March 22th, 2020 without any language restriction. We considered cross-sectional studies of HEV infection in animals in Africa. Study selection, data extraction, and methodological quality of included studies were done independently by two investigators. Prevalence data were pooled using the random-effects meta-analysis. This review was registered in PROSPERO, CRD42018087684.

**Results:**

Twenty-five studies (13 species and 6983 animals) were included. The prevalence (antibodies or ribonucleic acid [RNA]) of HEV infection in animals varied widely depending on biological markers of HEV infection measured: 23.4% (95% confidence interval; 12.0–37.2) for anti-HEV immunoglobulins G, 13.1% (3.1–28.3) for anti-HEV immunoglobulins M, and 1.8% (0.2–4.3) for RNA; with substantial heterogeneity. In subgroup analysis, the immunoglobulins G seroprevalence was higher among pigs 37.8% (13.9–65.4). The following HEV genotypes were reported in animals: Rat-HEV genotype 1 (rats and horses), HEV-3 (pigs), HEV-7 (dromedaries), and Bat hepeviruses (bats).

**Conclusions:**

We found a high prevalence of HEV infection in animals in Africa and HEV genotypes close to that of humans. Some animals in Africa could be the reservoir of HEV, highlighting the need of molecular epidemiological studies for investigating zoonotic transmission.

**Supplementary Information:**

The online version contains supplementary material available at 10.1186/s12917-021-02749-5.

## Background

Worldwide, hepatitis E virus (HEV) is a major cause of acute hepatitis in humans. HEV belongs to the family of Hepeviridae and consists of a non-segmented, non-enveloped and single-stranded ribonucleic acid (RNA) [1, 2]. This family is divided into the two genera: *Orthohepevirus* and *Piscihepevirus* [1, 3]. Four species designated as *Orthohepevirus A* to *D* are found in the genus *Orthohepevirus* [3]. *Orthohepevirus A* contains eight genotypes (HEV-1 to HEV-8) [1, 4]. HEV-1 and HEV-2 are exclusively detected in humans. These two genotypes are responsible for large hepatitis E outbreaks described in developing regions like Africa and Asia. HEV-3 and HEV-4 are present in humans and other animals, and are the main cause of sporadic infection among humans in developed countries. These two genotypes are considered zoonotic, and pigs and other animal species are reservoir of viruses infecting humans [5]. HEV-5 and HEV-6 have been identified in japanese wild boars [6]. HEV-7 has been described recently in an immunocompromised transplant patient and in dromedary camels [7, 8]. HEV-8 was detected recently in Bactrian camels in China [4]. *Orthohepevirus B* contains four subtypes (I–IV) of avian viruses identified mainly in domestic chicken. Two genotypes detected in rats (HEV-C1) and carnivores (HEV-C2) belong to *Orthohepevirus C*. Different bat species represent the animal reservoirs for *Orthohepevirus D* strains [1, 5, 9, 10]. *Piscihepevirus A* identified in cutthroat trout and related species is only one species described in the genus *Piscihepevirus* [1].

In 2010, one-third of the world’s population has been infected with HEV [11]. A case-fatality rate of 1–4% in the general population might reach 30% in pregnant women infected with HEV-1. In addition, chronic disease courses could be observed in immunocompromised transplant patients infected with HEV-3 [12, 13]. HEV causes large outbreaks and sporadic cases of acute hepatitis [14]. There is heterogeneity in the distribution of HEV as cause of acute hepatitis; with HEV responsible for more than 50% of the acute hepatitis infections in some countries like India, 15–20% in Eastern-Oriental countries, and 25% in Africa [15].

The systematic review published by Kim and colleagues in 2014 provided an overview of the epidemiology of HEV infection in humans in Africa. Since 1979, 17 HEV outbreaks have been reported, around once every year in Africa; causing a reported 35,300 cases with 650 deaths. Three HEV genotypes (HEV-1, 2 and 3) have been detected in humans in Africa and it appears that HEV-1 is most prevalent than HEV-2 and HEV-3 [14]. Animals might therefore play a role in the transmission of HEV in Africa as in developed countries. However, evidence of zoonotic transmission in Africa is not well established. Therefore, better knowledge of the epidemiology of HEV in animals in Africa can help to understand the epidemiology among humans and consequently help to design better strategy to curb the burden of HEV infection in Africa through interventions in animals and on interaction between humans and animals.

The objective of this study was to describe the epidemiology of HEV infection in animals in Africa based on a systematic review and meta-analysis. We specifically aimed to summarize data on the seroprevalence and viral prevalence of HEV infection in animals in Africa. We also aimed to identify different HEV genotypes found in animals in Africa, to investigate whether animal in Africa might represent a HEV reservoir.

## Methods

### Design

This systematic review with meta-analysis was registered in PROSPERO, CRD42018087684 and the protocol was published in a peer-review journal [16]. This review was reported according to PRISMA guidelines [17]. This systematic review and meta-analysis was conducted as recommended in the Joanna Briggs Institute reviewer’s manual for prevalence and incidence review [18].

### Criteria for considering studies for this review

#### Types of participants

We considered animals living in Africa regardless of age and sex. We excluded studies performed in laboratory animals, studies conducted in animals imported in the Africa continent, studies in which sample were not directly collected in animals (like feces in the breeding areas), and studies reporting HEV cases imported from outside Africa.

#### Types of outcomes

- HEV prevalence based on the biological markers including serological markers like immunoglobulins (Ig) G and Ig M or molecular markers like HEV RNA measured by reverse transcriptase polymerase chain reaction.
- HEV genotypes identified by molecular techniques.

#### Types of studies

We considered only cross-sectional studies.

### Search strategy and identification of studies

We searched Medline through PubMed, Exerpta Medica Database, African Journals Online, and Africa Index Medicus from January 1st, 2000 to March 22th, 2020 without any language restriction. We manually searched the reference list of all relevant articles and reviews to identify additional articles. The full search strategy in PubMed is available in the published protocol [16]. This search strategy was built according to PRESS guidelines [19]. In brief, text words and medical subject heading terms; and their variants were used. These terms included “Hepatitis E”, “Africa”, and individual names of African countries and regions; with a filter for animals.

Two review authors independently read titles and abstracts of the identified references and eliminated obviously irrelevant studies. These two review authors independently retrieved full-text articles for the references that remained, and examined potentially relevant studies, using the predetermined inclusion criteria. Existing disagreements were solved by a third review author.

### Data extraction and management

Two review authors independently extracted data from the included studies. Data were double checked by a third author. Data extracted included name of first author, year of publication, period of recruitment, types of genotypes, site of recruitment, sampling method, species, number of animals examined, number of animals infected with hepatitis E virus, ascertainment of HEV infection, age distribution, and proportion of male animals. We extracted data by animal species even coming from a unique study considering therefore each species in each study as the unit of analysis.

### Assessment of the methodological quality

An adapted version of the risk of bias tool for prevalence studies developed by Hoy and colleagues has been used to evaluate the methodological quality of included studies [20]. The defined items were scored with 0 for ‘No’ and 1 for ‘Yes’. The total score of each included study was calculated by the sum of its items. Methodological quality was considered as low, moderate, and high for scores of 0–4, 5–7 and 8–10, respectively. Two review authors assessed the methodological quality and disagreements were solved through a consensus or by an arbitration of a third review author.

### Data synthesis and analysis

Data analysis was conducted using the “*meta*” packages of the *R* statistical software (version 3.6.2, The *R* Foundation for statistical computing, Vienna, Austria). The prevalence of HEV infection was recalculated on the basis of numerators and denominators provided by individual studies. To minimize the effect of studies with extremely small or extremely large prevalence estimates on the overall estimate, the variance in study-specific prevalence was stabilized with Freeman-Tukey arc-sine transformation before pooling the data with the random effects meta-analysis model [21]. Heterogeneity was assessed by the chi-square test on Cochran’s Q test [22], which was quantified by I^2^ values, assuming I^2^ values of 25, 50 and 75% respectively representative low, medium and high heterogeneity [23]. When substantial heterogeneity was detected (I^2^ > 50%), we performed subgroup analysis by animal species to investigate possible sources of heterogeneity (for studies with 10 more animals by species). The Egger test was performed to assess the presence of publication bias and selective reporting [24]. A *p* value < 0.10 was considered indicative of a statistically significant publication bias. We synthesized data on genotypes in the narrative format.

## Results

### The review process

**Fig. 1:**
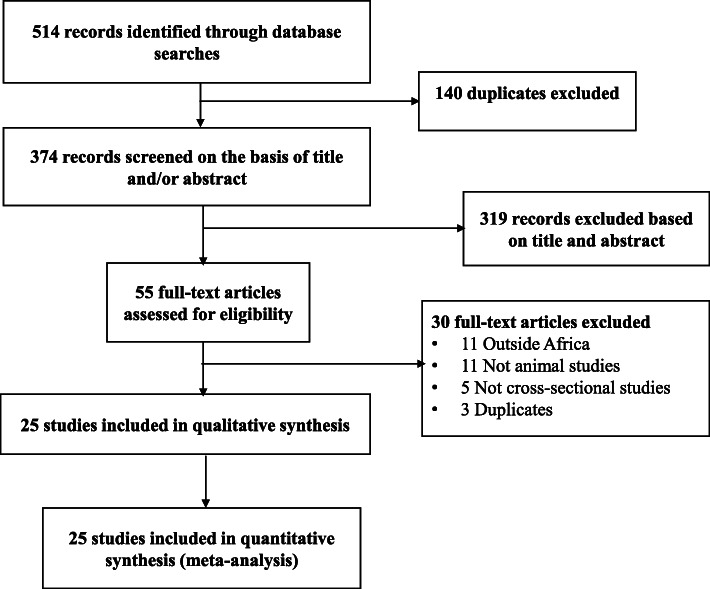
The review process

Initially, a total of 514 records were identified. One hundred and forty duplicates were removed. After screening titles and abstracts, 319 records were found irrelevant and then excluded. Agreement between review authors on abstract selection was high (Kappa = 0.85, *p* <  0.001). Full-texts of the remaining 55 records were scrutinized for eligibility, among which 30 were excluded. Finally, 25 studies were retained (Fig. 1) [8, 25–48].

### Characteristics of included studies

Studies were published between 2006 and 2020. Animals were included in studies between 1983 and 2018. Nine, five, and three studies were from rural, urban and both settings. Eight studies did not specify the setting. Sampling was consecutive and random in 13 and 12 studies respectively. Data were collected prospectively and retrospectively in 20 and five studies respectively. Data were from 19 countries. Five (20%) studies had low, 16 (64%) moderate, and four (16%) high risk of bias (Supplementary Table 1).

### Prevalence of HEV infection in animals in Africa

In total, data were from 6983 animals including 13 species (Bats (*n* = 1417), Buffaloes (*n* = 57), Chicken (*n* = 1), Cows and Beefs (*n* = 74), Donkeys (*n* = 47), Dromedaries (*n* = 1483), Ducks (*n* = 1), Goats and Sheep (*n* = 404), Horses (*n* = 200), Monkeys (*n* = 173), Pigs (*n* = 2721), Rabbits (*n* = 173), and Rats (*n* = 43)).

The prevalence of HEV infection in animals varied widely depending on biological markers: 23.4% (95% confidence interval; 12.0–37.2) for anti-HEV IgG, 13.1% (3.1–28.3) for anti-HEV IgM, and 1.8% (0.2–4.3) for HEV RNA with substantial heterogeneity (Table 1). The 95% prediction interval was wide for anti-HEV IgG and anti-HEV IgM. There was no publication bias (Table 1) as indicated by funnel plots for anti-HEV IgG and RNA (Supplementary Figs. 1 and 2).

**Table 1:** Summary statistics of meta-analysis prevalence of hepatitis E virus infection in animals in Africa

Biological markers	Prevalence, % (95% confidence interval)	Studies, n	Animals, n	Heterogeneity			*P* Egger test
				95% Prediction interval	I^**2**^, %	***P*** value	
Ribonucleic acid	1.8 (0.2–4.3)	14	4621	0.0–17.0	94.6	< 0.0001	0.174
Immunoglobulins M	13.1 (3.1–28.3	5	1174	0.0–81.1	97.6	< 0.0001	0.755
Immunoglobulins G	23.4 (12.0–37.2)	14	3016	0.0–83.3	98.6	< 0.0001	0.450

**Fig. 2:**
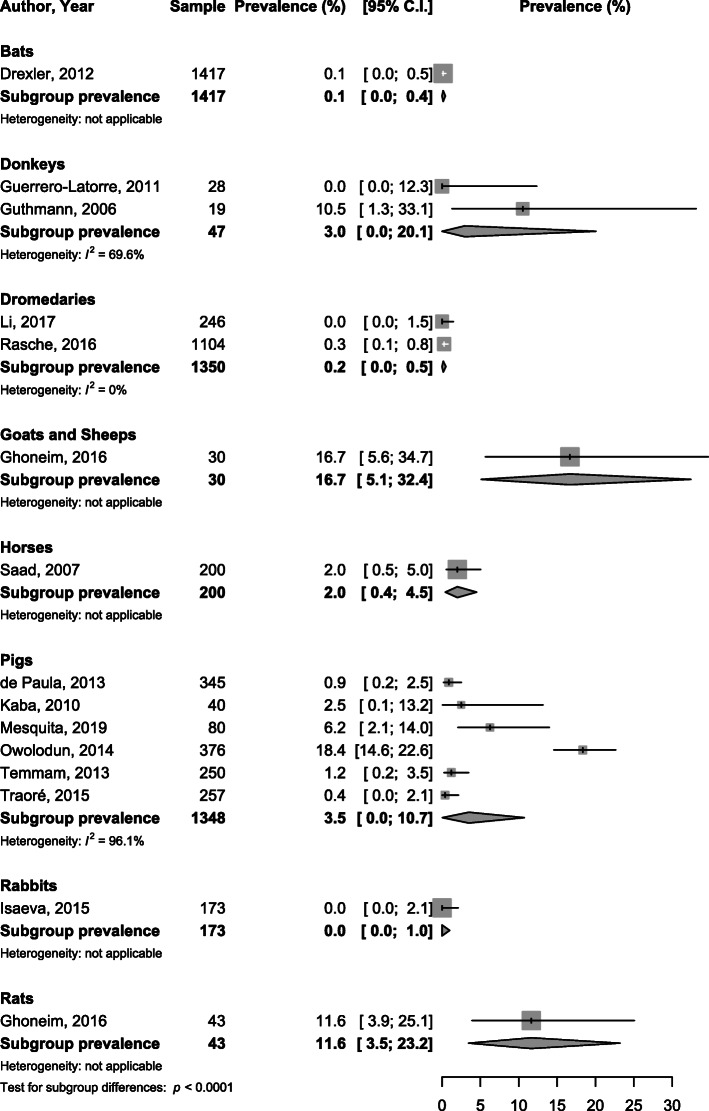
Meta-analysis of viral prevalence of HEV infection in animals in Africa

**Fig. 3:**
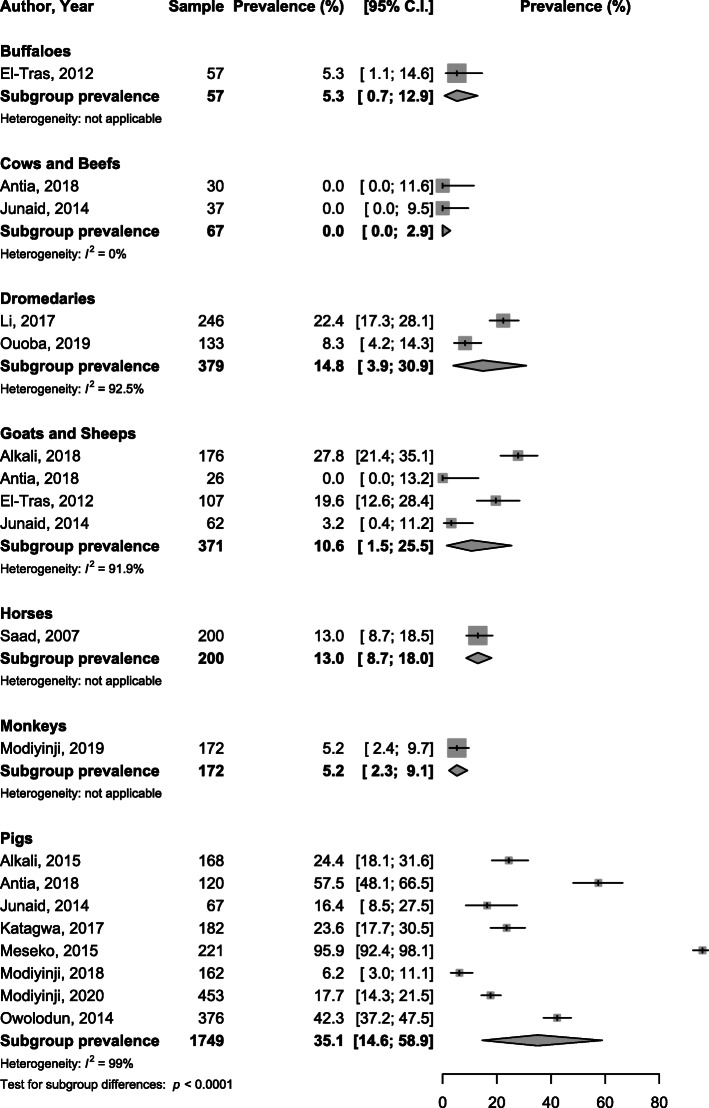
Meta-analysis immunoglobulins G seroprevalence of HEV infection in animals in Africa

**Fig. 4:**
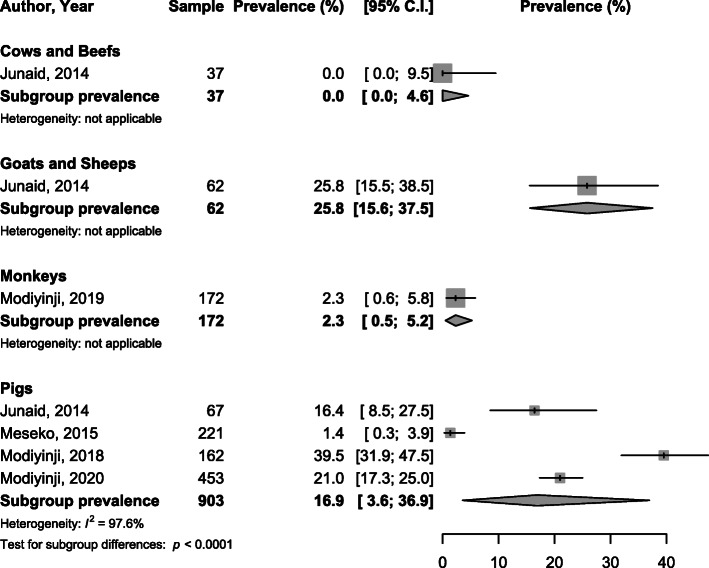
Meta-analysis immunoglobulins M seroprevalence of HEV infection in animals in Africa

When considering subgroup analysis by specie, the viral HEV prevalence varied from 0% in rabbits to 16.7% in goats and sheep with significant difference between species considering RNA, *p* <  0.0001 (Fig. 2). The IgG seroprevalence varied from 0.0% in cows and beefs to 35.1% in pigs with significant difference between species, *p* <  0.0001 (Fig. 3). IgM seroprevalence varied from 0% in cows and beefs to 25.8% in goats and sheep, *p* <  0.0001 (Fig. 4).

### HEV genotypes in animals in Africa

The following four HEV genotypes were reported by 10 studies: rat-HEV genotype 1, Bat hepevirus, HEV-3, and HEV-7 (Table 2). Seven studies demonstrated the presence of HEV-3 in pigs in Cameroon, Democratic Republic of Congo, Nigeria, São Tomé and Príncipe, Madagascar and Burkina Faso. Rat-HEV genotype 1 was found in rats and horses in Egypt. HEV-7 was found in dromedaries in Somalia and Kenya. Additionally, bat hepevirus was found in bats in Ghana and Gabon (Table 2).

**Table 2:** Hepatitis E genotypes in animals in Africa

Genotypes	Studies	Animals	Countries
**Rat-HEV genotype 1**	Ghoneim, 2016 [30]	Rats	Egypt
	Saad, 2007 [42]	Horses	Egypt
**HEV-3**	de Paula, 2013 [27]	Pigs	Cameroon
	Modiyinji, 2020	Pigs	Cameroon
	Traoré, 2015 [44]	Pigs	Burkina Faso
	Kaba, 2010 [35]	Pigs	Democratic Republic of the Congo
	Temmam, 2013 [43]	Pigs	Madagascar
	Owolodun, 2014 [41]	Pigs	Nigeria
	Mesquita, 2019 [38]	Pigs	São Tomé and Príncipe
**HEV-7**	Rasche, 2016 [8]	Dromedaries	Somalia; Sudan; Egypt; Kenya
**Bat hepevirus**	Drexler, 2012 [28]	Bats	Ghana; Gabon

## Discussion

This systematic review with meta-analysis reveals that the prevalence of HEV infection in animals varied widely depending on biological markers: 23.4% with IgG, 13.1% with IgM, and 1.8% with RNA. We also found a substantial heterogeneity in the overall IgG and IgM seroprevalence and viral prevalence that was explained by difference in the HEV distribution among different animal species. Pigs presented the highest IgG seroprevalence of 35.1% followed by dromedaries, cows and beefs, and goats and sheep with IgG seroprevalence between 10 and 15%. Donkeys, pigs, rats, and goats and sheep presented a viral prevalence of more than 3%. Pigs, goats, and sheep presented an IgM seroprevalence higher than 10% compared to others. Four genotypes were identified in animals in Africa: rat-HEV genotype 1, HEV-3, HEV-7 and bat hepevirus.

The seroprevalence (IgM 16.9% and IgG 35.1%) and viral prevalence (3.5%), we found among pigs overlap prevalence estimates from other countries outside Africa [49]. Like in Africa, there is a wide variation in IgG seroprevalence in pigs living in other regions including North America (35–59%), Oceania (72–91%), South America (23–64%), Europe (20–97%), and Asia (9–100%) [49]. In a recent systematic review and meta-analysis reported from Mainland China among pigs, the IgG seroprevalence was 66% (95% CI: 62–71) [50], higher to the IgG seroprevalence found in this study.

Among sheep and goats, studies in China reported a seroprevalence of IgG varying from 14.3 to 35.2% [51, 52], slightly higher than the finding of this study (10.6%). The same figure is found for viral prevalence where prevalence data from China (74.1%) were higher compared to prevalence in Africa (16.7%) and Europe (9.2%) [52, 53]. A study reported in the Middle East revealed that 1.5% (0.5–4.3) of the dromedaries showed evidence of the presence of HEV RNA in their blood samples [54, 55]. The viral prevalence found in this study was 0.2% (0.0–0.5), slightly lower compared to data from Middle East. Detection of HEV RNA in dromedary in Africa was done from blood samples while in Middle East, laboratory analysis was performed on stool. Evidence support that HEV presence in fecal excretion lasts longer than viremia [56]. A study performed in 12 European countries between 2012 and 2015 showed a viral prevalence of rat-HEV genotype 1 in 12.4% of 508 rats [55], higher than that we found (11.6%) in this study. This European study used two molecular methods (real-time and conventional PCR) that target different HEV Orthohepevirus species whereas the Egyptian study used only one method (conventional PCR). The high sensitivity of the real-time PCR has been demonstrated in studies and may explain the differences observed in both studies [57].

Among cows, studies conducted in China between 2015 and 2018 showed a viral prevalence that varied between 0 and 37.1% [58–60]. In a study conducted in Belgium, the prevalence was 0% for HEV RNA and for IgG anti-HEV [61]. We also found an IgG seroprevalence of 0% from two studies. Among buffaloes, study conducted in Lao People’s Democratic Republic in 2015 showed an IgG seroprevalence of 20% [62], higher than that we found in Africa (5.3%).

To date, HEV infection remains a global public health concern [15]. It is therefore important to implement strategies to curb the burden through public health education and intervention programs, improved clinical practice and innovation in research for this infection [63]. Since, this study demonstrated that some HEV genotypes found in animals were already found in humans, large-scale molecular epidemiological studies are needed in the continent to investigate any HEV zoonotic transmission. This zoonotic transmission was already demonstrated in other settings [2]. This study also shows that other animal species including chickens and wild boar, already identified as a reservoir of HEV are not yet studied in Africa. It is therefore necessary to perform investigations in these animals [64–69].

Although the rate of zoonotic transmission to humans and its public health importance remain unclear [70]; strategies are needed to prevent the potential zoonotic transmission, especially among individuals with direct contact with animals [71]. Since there is no specific treatment for HEV infection, public health policy makers should implement comprehensive public health measures, especially for high group risk individuals in African countries such as educating the farmers, slaughterers, butchers, forest workers, hunters, and veterinarians [72, 73]. A hepatitis E vaccine was licensed in China in 2012, and is currently the only hepatitis E vaccine available. Nonetheless, the World Health Organization did not issue a broad recommendation for its routine use outside of China [74]. Therefore, the development of an effective vaccine to vaccinate humans and also animals is urgently needed in order to minimize the risk of HEV zoonotic transmission [75]. Because cross-species transmission and host tropisms of zoonotic HEV genotypes are not yet understood, surveillance studies of swine and wildlife reservoirs should be conducted to identify all possible human exposure pathways [75].

This study has several limitations. First, we found substantial heterogeneity in the estimation of the overall prevalence of hepatitis E infection. We were able to identify some sources of heterogeneity including biomarkers considered and animal species. However, due to inconsistency in original studies, we were not able to investigate other sources of heterogeneity including population characteristics and variability in sensitivity and specificity of diagnostic methods in original studies. Second, African countries were not uniformly represented and can limit the generalizability of findings to the entire continent. Third, only 20% of the studies were assessed as having low risk of bias in their methodological quality suggesting that high quality epidemiological studies are needed. However, we were not able to perform sensitivity analysis including only studies with high methodological quality due to paucity of data. Despite these limitations, this study is, to the best of our knowledge, the first systematic review and meta-analysis on prevalence of HEV infection in animals in Africa. We also included the investigation of different HEV genotypes. Strengths include a comprehensive search strategy and involvement of two independent investigators in all stages of the review process to minimize bias. We did not have publication bias for all outcomes.

## Conclusions

We found a high prevalence of HEV infection in animals in Africa and HEV genotypes close to that of humans. As such, hepatitis E infection should deserve more attention from healthcare providers, researchers, policymakers and stakeholders from many sectors. Some animals in Africa could be the reservoir of HEV, highlighting the need of molecular epidemiologic studies for investigating zoonotic transmission.

## Supplementary Information

**Additional file 1: Supplementary Table 1**. Individual characteristics of included studies. **Supplementary Figure 1**. Funnel plot for publication bias of IgG seroprevalence among animals in Africa. **Supplementary Figure 2**. Funnel plot for publication bias of RNA prevalence among animals in Africa.

## Data Availability

The datasets used and analyzed during the current study are available from the corresponding author on reasonable request.

## Abbreviations

HEV: Hepatitis E virus
IgG: Immunoglobulins G
IgM: Immunoglobulins M
RNA: Ribonucleic acid
