# Supplementary material for: Epidemiology of hepatitis E virus infection in animals in Africa: a systematic review and meta-analysis
Source: BMC Vet Res. 2021 Jan 25;17:50. doi: 10.1186/s12917-021-02749-5 (PMC7831161; doi:10.1186/s12917-021-02749-5)

Epidemiology of hepatitis E virus infection in animals in Africa: a systematic review and meta-analysis

Abdou Fatawou Modiyinji, Jean Joel Bigna, Sebastien Kenmoe, Fredy Brice N. Simo, Marie A. Amougou, Marie S. Ndangang, Moise Nola, and Richard Njouom

APPENDIX

Supplementary Table 1. Individual characteristics of included studies 2

Supplementary Figure 1. Funnel plot for publication bias of IgG seroprevalence among animals in Africa 4
Supplementary Figure 2. Funnel plot for publication bias of RNA prevalence among animals in Africa 4

Supplementary Table 1. Individual characteristics of included studies

Study	Area	Timing	Period of recruitment	Country	Population	Sample	Sampling	Age range	Sample type	Diagnostic technic	Risk of bias
Alkali, 2015	Undefined	prospective	undefined	Nigeria	Pigs	168	Random	undefined	blood	ELISA	Moderate
Alkali, 2018	Undefined	prospective	2017	Nigeria	Goats and Sheep	176	Random	undefined	blood	ELISA	Moderate
Antia, 2018	Urban	prospective	2016	Nigeria	Pigs, Goats and Sheep	176	Random	undefined	blood	ELISA	Moderate
de Paula, 2013	Urban	prospective	2012	Cameroon	Pigs	345	Consecutive	6 months-3 years	liver	Conventional RT-PCR	Moderate
Drexler, 2012	Undefined	prospective	2002-11	Ghana; Gabon	Bats	1417	Consecutive	undefined	blood; stool; Liver	Conventional RT-PCR	Low
El-Tras, 2012	Rural	prospective	2010-11	Egypt	Buffaloes, Cows and Beefs, Goats and Sheep	185	Consecutive	undefined	blood	EIA	High
Ghoneim, 2016	Rural	prospective	undefined	Egypt	Rats, Goats and Sheep	73	Consecutive	undefined	blood; stool	ELISA; Conventional RT-PCR	Moderate
Guerrero-Latorre, 2011	Rural	prospective	2009	Chad	Donkeys	28	Consecutive	undefined	stool	Conventional RT-PCR	Moderate
Guthmann, 2006	Rural	prospective	2004	Sudan	Donkeys	19	Random	undefined	blood; stool	Conventional RT-PCR	Low
Isaeva, 2015	Rural	prospective	undefined	Egypt	Rabbits	173	Random	2-10 months	stool	Conventional RT-PCR	Low
Junaid, 2014	Rural	prospective	2012	Nigeria	Pigs, Cows and Beefs, Goats and Sheep	166	Random	undefined	blood	ELISA	Moderate
Kaba, 2010	Rural	prospective	undefined	Democratic Republic of the Congo	Pigs	40	Consecutive	2-4 months	stool	real time RT-PCR	Moderate
Katagwa, 2017	Undefined	prospective	2015	Uganda	Pigs	182	Random	undefined	blood	ELISA	Moderate
Li, 2017	Undefined	prospective	2013	Ethiopia	Dromedaries	246	Consecutive	1-13 years	blood	ELISA; Conventional RT-PCR	Moderate
Meseko, 2015	Urban	prospective	2012	Nigeria	Pigs	221	Consecutive	undefined	blood	ELISA	High
Mesquita, 2019	Undefined	Retrospective	2011	São Tomé and Príncipe	Pigs, Chicken, Cows and Beefs, Duck, Goats and Sheep, Monkeys	93	Random	undefined	stool	real-time RT-PCR, nested RT-PCR	Moderate
Modiyingi, 2019	Rural	Retrospective	1997-2006	Cameroon	Monkeys	172	Random	Undefined	Blood	ELISA	Low
Modiyingi, 2020	Urban, rural	prospective	2017-2018	Cameroon	Pigs	453	Random	Undefined	Blood; stool	ELISA; RT-PCR	Low
Modiyinji, 2018	Undefined	prospective	2012	Cameroon	Pigs	162	Random	undefined	blood	ELISA	Moderate
Ouoba, 2019	Rural	prospective	2015	Burkina Faso, Mali, Niger	Dromedaries	133	Random	0.1-18 years	blood	ELISA	Moderate

Owolodun, 2014	Urban; rural	Retrospective	2009; 2011-12	Nigeria	Pigs	376	Consecutive	1 month-3 years	blood; stool	ELISA; real timeRT-PCR; Conventional RT-PCR	High
Rasche, 2016	Undefined	Retrospective	1983-2015	Somalia; Sudan; Egypt; Kenya	Dromedaries	1104	Consecutive	undefined	blood	ELISA; Conventional RT-PCR	Moderate
Saad, 2007	Urban; rural	prospective	1999	Egypt	Horses	200	Consecutive	0.2-12 years	blood	ELISA; EIA; Conventional RT-PCR	Moderate
Temmam, 2013	Urban	Retrospective	2008-11	Madagascar	Pigs	250	Consecutive	more than 6 months	blood; liver	EIA; ELISA; Conventional RT-PCR	High
Traoré, 2015	Urban	prospective	2012-13	Burkina Faso	Pigs	257	Consecutive	6-18 months	blood; liver	ELISA; Conventional RT-PCR	Moderate

Supplementary Figure 1. Funnel plot for publication bias of IgG seroprevalence among animals in Africa

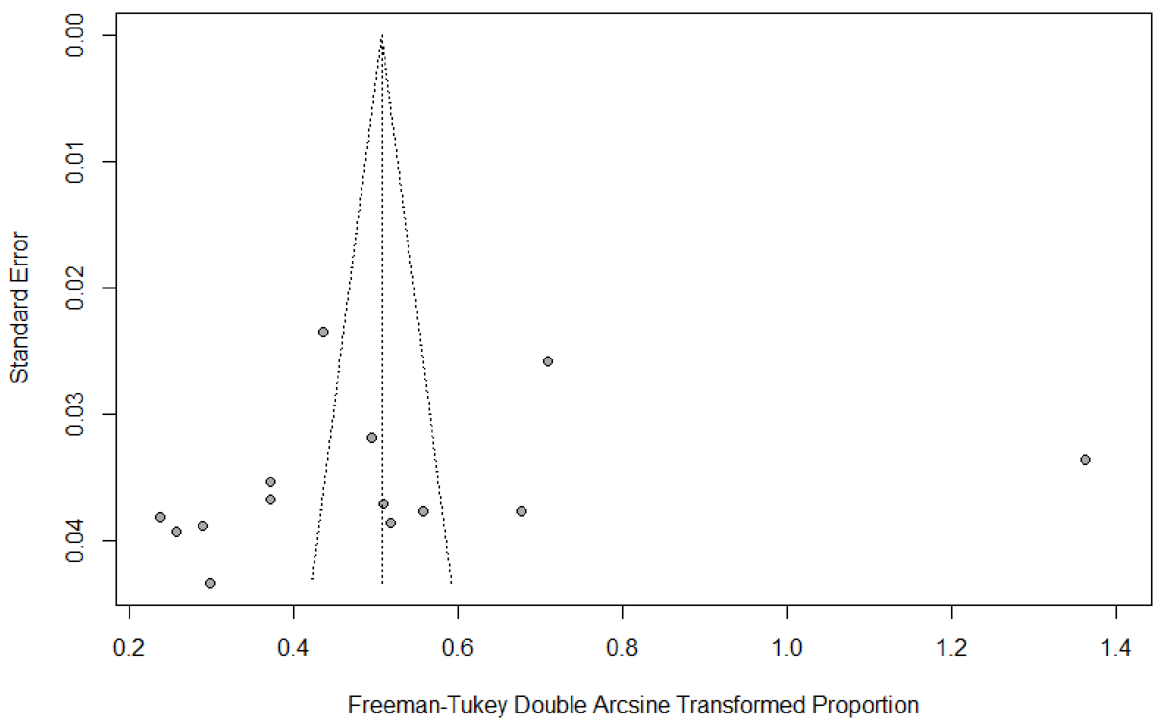

Supplementary Figure 2. Funnel plot for publication bias of RNA prevalence among animals in Africa

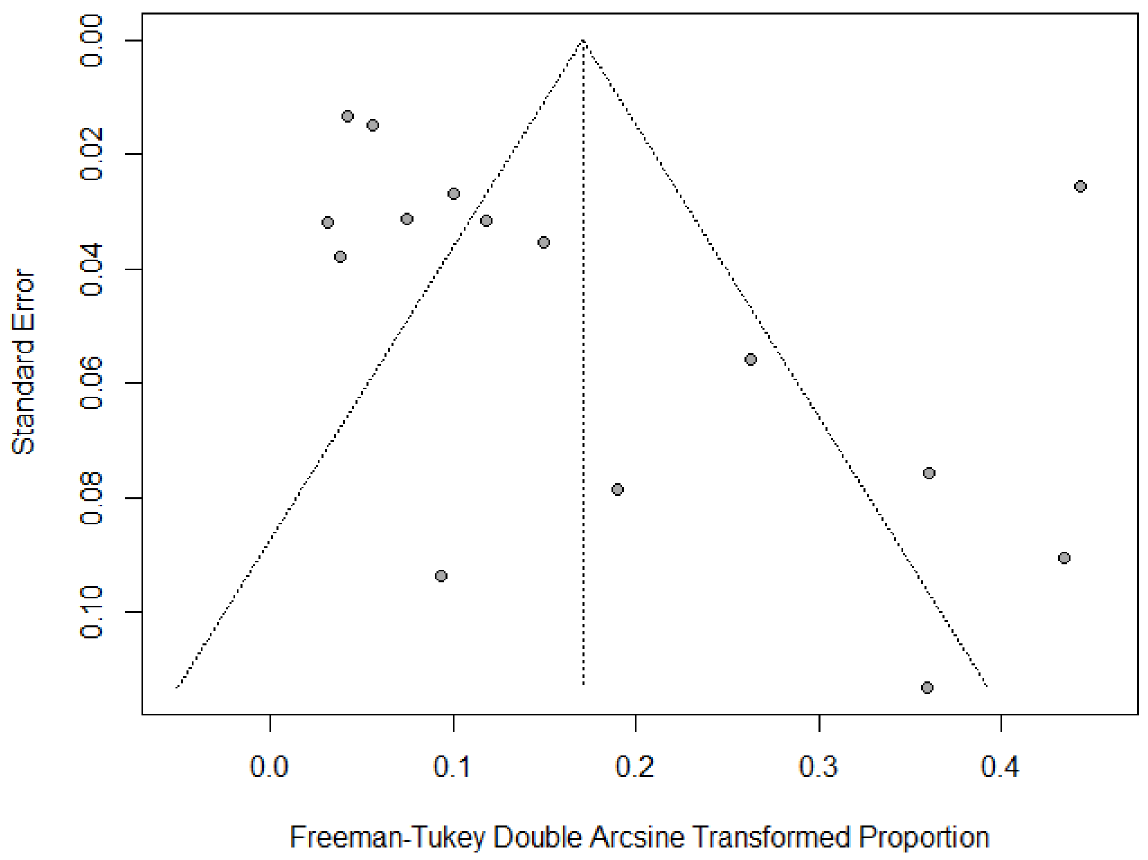

Supplement: Supplementary file 1 — Additional file 1: Supplementary Table 1. Individual characteristics of included studies. Supplementary Figure 1. Funnel plot for publication bias of IgG seroprevalence among animals in Africa. Supplementary Figure 2. Funnel plot for publication bias of RNA prevalence among animals in Africa. [file 12917_2021_2749_MOESM1_ESM.pdf]
